# Newcomer knowledge, attitudes, and beliefs about human papillomavirus (HPV) vaccination

**DOI:** 10.1186/s12875-020-01360-1

**Published:** 2021-01-09

**Authors:** Lindsay A. Wilson, Amanda M. L. Quan, A. Brianne Bota, Salima S. Mithani, Michelle Paradis, Cindy Jardine, Charles Hui, Kevin Pottie, Natasha Crowcroft, Kumanan Wilson

**Affiliations:** 1 Clinical Epidemiology Program, Ottawa Hospital Research Institute, Administrative Services Building, Box 684, 1053 Carling Avenue, Ottawa, ON K1Y 4E9 Canada; 2 Faculty of Health Sciences, University of the Fraser Valley, 45190 Caen Ave., Chilliwack, BC V2R0N3 Canada; 3 Faculty of Medicine, University of Ottawa, 451 Smyth Rd, Ottawa, ON K1H 8M5 Canada; 4 Bruyère Research Institute, 85 Primrose Ave, Ottawa, ON K1R 6M1 Canada; 5 Public Health Ontario, 480 University Avenue, Suite 300, Toronto, Ontario M5G Canada; 6 Department of Medicine, University of Ottawa, 451 Smyth Rd, Ottawa, ON K1H 8M5 Canada

**Keywords:** Human papillomavirus (HPV), Vaccine, Newcomers, Barriers to care, Canada

## Abstract

**Background:**

Human Papillomavirus (HPV) is the most common sexually transmitted infection in Canada and around the world. Vaccination is an effective prevention strategy, but uptake is low, especially among newcomers to Canada. We sought to understand newcomers’ knowledge, attitudes, and beliefs (KAB) on HPV and HPV vaccination and their role in HPV vaccine acceptance.

**Methods:**

Newcomers were defined as individuals born outside Canada, (i.e., individuals born in a different country, the majority of whom are immigrants or refugees, but also includes students and undocumented migrants). Eligible participants were newcomers, aged 16 or older and who could read or write in English, French or Arabic. Surveys were administered in two community health centres in Ottawa, Canada that primarily engage with newcomer populations. Follow-up interviews were conducted either at the community health centre or over the phone, depending on participants’ preferences.

**Results:**

Fifty participants completed the survey, the majority of whom were women (74%) and spoke Arabic as their first language (54%). Seven participants completed supplemental interviews to complement their survey responses. The majority (70%) of participants had not previously heard of HPV. Less than half (46%) knew that the vaccine is effective in preventing certain types of cancer; nearly 40% incorrectly believed the vaccine could cure HPV. Qualitative interviews supported the survey findings.

**Conclusions:**

Despite a lack of HPV knowledge due to cultural and language barriers, there is still a strong desire among newcomers to receive the vaccine, especially when accompanied by a physician recommendation. Cultural and language-appropriate resources are needed to help newcomers make informed vaccination decisions and promote HPV vaccine uptake.

**Supplementary Information:**

The online version contains supplementary material available at 10.1186/s12875-020-01360-1.

## Background

Human Papillomavirus (HPV) is the most common sexually transmitted infection in Canada and around the world [1]. It is estimated that 75% of sexually active Canadians will have at least one HPV infection in their lifetime [2]. HPV infection is the cause of nearly all cervical cancers [1] and can also result in numerous other genital and oropharyngeal cancers [3]. Vaccination has proven to be an effective primary prevention strategy to protect against HPV infection and HPV-associated pre-cancerous lesions [4–6].

Publicly-funded, predominantly school-based HPV vaccination programs were introduced in Canada in 2006, and all provinces and territories now offer publicly-funded HPV vaccination for both boys and girls [7]. Despite the effectiveness and widespread availability of the HPV vaccine, HPV vaccination uptake is estimated to be only 56% in Canada, well below the goal of > 80% coverage [8]. Low uptake was also observed in a systematic review evaluating vaccine uptake by parents for their children across 15 countries with HPV vaccination programs, including Canada and the US. Gender was found to be a factor in determining uptake, with vaccination among girls occurring twice as often as among boys [8–10].

A systematic review evaluating HPV vaccine uptake in Canada also found vaccination rates to be higher in females (57%) compared to males (47%) and higher in participants 18 years of age or younger (67%) [8]. Vaccine cost was found to be a significant barrier to vaccine uptake in Canada, where HPV vaccination costs are only covered for school-aged children (< 26 years of age for most private insurance plans). Specifically, uptake was less than 14% when participants were required to pay out of pocket [11]. However, healthcare provider recommendation appears to play an important role in encouraging parents to accept the vaccine for their children [9, 10].

One group for whom it is difficult to ascertain HPV vaccination uptake information is newcomers to Canada. More than 200,000 newcomers arrive in Canada each year [12]. These numbers primarily comprise immigrants- individuals choosing to settle in a country other than the one in which they were born - and refugees but also include students and undocumented migrants. Newcomers may not be comfortable answering questions about their vaccination history due to language barriers or cultural norms about discussing sexual health [14]. Immigrants and refugee populations in Canada have been found to have lower screening rates for cervical cancer and higher rates of HPV infection, putting them at increased risk of morbidity and mortality from HPV infection and associated cancers [15–17].

The objective of this study was to better understand the knowledge, attitudes and beliefs (KAB) of newcomers surrounding HPV and the HPV vaccine. The study was guided by the following research questions:

1. What knowledge do newcomers possess regarding HPV and the HPV vaccine?
2. What beliefs and values do newcomers possess regarding HPV and the HPV vaccine?
3. What barriers do newcomers face when accessing HPV vaccination?

## Methods

### Study setting

This study was conducted in Ottawa, Canada, where immigrants and refugees make up over 20% of the population [12]. Ottawa-Gatineau has the fifth highest number of foreign-born people in Canada [13]. Among recent immigrants, the most-commonly spoken language is Arabic and the greatest proportion of people originate from Asia (44%) and Africa (24%) [13]. The HPV vaccine is offered in publicly-funded, school-based vaccination programs to all students in Ontario when they are in Grade 7 (age 11–12). This study and all study materials were approved by the Ottawa Health Science Network Research Ethics Board (OHSN-REB #20170913-01H). All participants provided informed written consent prior to participating in any study-related activities. Participants’ names and contact information were stored separately from their responses in order to ensure confidentiality.

### Participant recruitment

Eligible participants were:

- Young Adults: Between the ages of 16 and 27 (the upper age limit for which many insurance plans will cover HPV vaccination in Ontario), any gender, did not have children, and were either newcomers or the children of newcomers.
- Caregiver: Over the age of 18, any gender, born outside of Canada, and had one or more children under the age of 18.

Participants were recruited from two local community health centres (CHCs) in Ottawa that offer a variety of primary healthcare services to patients, including vaccination, and have considerable contact with Ottawa’s newcomer population. Identifying appropriate participants was facilitated by gatekeepers at each of the clinics. Those deemed eligible to participate by gatekeepers were introduced to the research staff who provided further details on the study and obtained informed written consent.

### Survey instrument

The survey was developed in-house based on findings from our previously published systematic review of barriers to vaccination among newcomers [14]. The survey had not been previously validated but was reviewed and revised in collaboration with staff from the CHCs used as recruitment sites and with the healthcare providers. The caregiver and young adult survey contained questions on demographics, immigration status, knowledge of HPV and HPV vaccine, and vaccine acceptance. There were a total of 14 questions with multiple prompts.

### Data collection

Surveys were conducted between May and July 2018. All participants who had previously heard of HPV and the HPV vaccine were asked to complete 11 additional questions to evaluate their level of HPV knowledge. Participants were also asked questions about vaccine acceptance. The survey was available in English, French, and Arabic. Participants 18 years of age and older who had children of any age were offered the “caregiver” version of the survey. Participants between the ages of 16–27 years who did not have children received a “young adult” version of the survey containing a restricted set of questions without references to decision-making for one’s children. All surveys were conducted in-person and returned to the research staff on the same day they were completed. The survey was stopped when a maximum of 50 participants were reached.

Semi-structured interviews were held with seven participants out of the fifty who completed the survey and who consented to being re-contacted for the purposes of the study. These interviews took place between June and August 2018, and were administered in English, French, or Arabic by research staff trained in qualitative research. An interpreter was provided to translate between English and Arabic. Interviews were conducted either in-person or over the phone, according to the participant’s preference. All interviews were audio-recorded, transcribed verbatim and analysed for emergent themes using a thematic analysis approach [18]. Interviews lasted an average of 25 min, with a range of 15 to 50 min. The interviewees were asked questions about their knowledge, uptake and beliefs around HPV and HPV vaccination (Supplemental Table 1).

### Data analysis

Survey data were analyzed using STATA 15.1 and was summarized using descriptive statistics. Chi-square tests were used to identify differences in HPV and HPV vaccine awareness and willingness to vaccinate across age groups, gender and level of education. Age was categorized into three distinct strata: 16–27, 28–44, and ≥45. Level of education was categorized into three distinct strata: completed high school or less, incomplete college or university and completed college or university. Statistical significance was set at *p* ≤0.05 [17–20] (18)(18)(18). A thematic analysis approach [18] was used to qualitatively analyze the survey open responses and interviews.

## Results

### Participant characteristics

Surveys were offered to those deemed to fit the eligibility criteria by the CHC clinicians. All potential participants deemed eligible by the clinician agreed to participate in the survey (*n* = 50). Participant characteristics are summarized in Table 1. Twenty-seven participants (54%) chose to complete the survey in Arabic, 11 in English (22%) and 12 in French (24%).

**Table 1:** Descriptive characteristics of 50 newcomer survey respondents in Ottawa, Canada

Characteristic	Respondents (*N* = 50)	%
Survey Language		
English	11	22.0
French	12	24.0
Arabic	27	54.0
Gender		
Female	38	76.0
Male	12	24.0
Age		
16–27^a^	13	26.0
28–44	19	38.0
45+	15	30.0
Prefer not to say	3	6.0
Religion		
Christian	17	34.0
Muslim	30	60.0
Agnostic	1	2.0
None	2	4.0
Immigrant Status		
Immigrant	20	40.0
Refugee	19	38.0
Other	9	18.0
Prefer not to say	2	4.0
Marital Status (*n* = 41)^b^		
Single	3	6.0
Married	34	68.0
Widowed	3	6.0
Prefer not to say	2	4.0
Number of children (*n* = 41)		
1	4	9.8
2	12	29.3
3	11	26.8
4	5	12.2
5+	9	22.0
Region of Origin		
Sub-Saharan Africa	18	36.0
Middle East and North Africa	29	58.0
Other	3	6.0

^a^ Nine of the participants in the 16–27 category completed the young adult variation of the survey, while the others had children and thus completed the adult version

^b^ Sample size reflects number of participants who completed the Caregiver version of the survey; Young Adults were not asked for their marital status

Participants from 18 different countries responded. The majority of participants were originally from the Middle East and North Africa (*n* = 29, 58%) region. Thirty-eight (76%) of the participants were female and 24% were male. Islam and Christianity were the two religions with which participants most commonly identified (60 and 34%, respectively). Nine (18%) of the participants completed the young adult survey, whereas the remaining 41 (82%) respondents completed the caregiver survey. The average age of the young adult group was 22.4 ± 3.7 years (range 17–27 years) and the caregiver group was 32.0 ± 10.1 (range: 21–57) years. The caregivers had between 1 and 11 children (mean 3.5 ± 2.1) ranging in age from 0 to 31 years. The majority of the caregiver population were married (*n* = 34, 68%). 48% (*n* = 20) of the caregivers had completed some form of postsecondary education.

Seven (5 females, 2 male) of the caregiver participants agreed to be contacted for follow-up and completed the interviews. Four of the seven interviewees were from Africa and all interviews were conducted in English or French. Individuals who participated in interviews had between 1 and 11 children. Four emergent themes were identified from the interviews and illustrative quotes are summarized in Table 2.

**Table 2:** Quotations illustrating emergent study themes from interviews with seven newcomers to Ottawa, Canada

Theme	
**1. Lack of HPV Knowledge**	
***Low Knowledge***	
*Interviewer: Have you heard of HPV before? If yes, what do you know about it?*“*Nothing” (Participant #3)*	
*“I think – where I come from, it’s not – we’re not – most people are not knowledgeable about it because I think it’s treated with – it’s not treated with as much attention as it should be given.” (Participant #1)* *“no, had never heard of it” - (Transcript 5)*	
***Misconceptions about HPV and the HPV vaccine***	
*“...they go to [the] swimming pool for example, they don’t have boyfriends or girlfriends, but because they go to public places, I’m concerned about that [HPV]... maybe with the public toilets they use. I want to make sure they’re protected. Public toilets, I’m very concerned about that.” (Participant #6)*	
**2. Willingness to accept vaccination**	
*Interviewer: “Do you believe that you would have your children vaccinated against HPV?* *“And my kids also, I would love them to get it when they get to the age. Yeah because you don’t play with your health, yeah. Health is wealth like they say.” (Participant #1)*	
*“They are very important [vaccines]. As, there is something said at home, I do not know if here we say it, it is better to prevent than cure. We warn like that.” (Participant #4)*	
**3. Access to vaccination**	
***Cost***	
*“They should cover it, that’s very sad. Most people those ages [*i.e.*, young adults no longer covered by the school-based programs], they don’t have a permanent job, they’re working here and there, they can’t afford that, it’s very hard for them. They should make it easier for them to protect themselves and then they won’t have to pay for them to be treated and treated for cancer.” (Participant #6)*	
*“Where I’ve come from, most vaccines are free [...] The government paid for them to pay for the – for the health workers at those administration boards, the recipients get it for free.” (Participant #2)*	
***Lack of time and healthcare provider recommendation***	
*Interviewer: “Did your doctor recommend the HPV vaccine for you (or your kids)”?* *“It’s very hard because when you go to the doctor, you have to limit your reason for going, you wait a lot of time and you have to have just one reason to be there, and it’s all a rush. There’s no time to mention that [HPV].” (Participant #6)*	
**4. Cultural norms**	
*“I believe this is a bit difficult to speak about …. Africans don’t talk a lot about sex with their children. If it’s with girls, no it is difficult, with boys I can see us talking a little bit, but not in depth. With girls it is very difficult.” (Participant #7)* *Interviewer: “So, do you think you would leave the choice to be vaccinated or not get vaccinated up to your daughter”?* *Participant #4: “Yes”.*	

### HPV knowledge

The caregiver and young adult responses to HPV and HPV vaccine knowledge and beliefs are summarized in Table 3. The majority (*n* = 35, 70%) of survey respondents had not previously heard of HPV; 3 young adults and 13 caregivers had heard of HPV and 2 preferred not to say. No significant associations were identified between age group (*X*^*2*^*(*4, *N* = 50)=1.2845, *p =* 0.536) or gender (*X*^*2*^*(*2, *N* = 50)=0228, *p =* 0.637) and awareness of HPV or age group (*X*^*2*^(4, *N* = 50)=0.5109, *p =* 0.775) or gender ((*X*^*2*^*(*2, *N* = 50)=0.082, *p =* 0.928) and awareness of HPV vaccine. Level of education was associated with awareness of HPV (*X*^*2*^(2, *N* = 41) =6.8742 *p = 0.032)* or HPV vaccine (*X*^*2*^(2, *N* = 41)=5.8011,*=0.055)*. When asked whether they had heard of the HPV vaccine one participant responded, *“I think – where I come from, most people are not knowledgeable about it because I think it’s treated with – it’s not treated with as much attention as it should be given.”*

**Table 3:** Knowledge and Beliefs around HPV and HPV vaccine

	Caregiver Survey response N (%)	Young Adult Survey Response N(%)	Overall Response N (%)
*Heard of HPV*	*N* = 41	*N* = 9	*N* = 50
Yes	13 (30%)	2 (22%)	15 (28%)
No	28 (65%)	7 (78%)	35 (70%)
Prefer not to say	0	0	2 (4%)
*Source of information*	*N* = 13	*N* = 2	*n* = 14
Doctor	2	0	2 (14%)
Nurse	0	0	0 (0%)
Family member	3	0	2 (14%)
Friend	1	1	1 (7%)
School	1		1 (7%)
Found their own information	6	1	6 (42%)
Other	2	0	2 (14%)
*Belief around HPV transmission*	*N* = 15	*N* = 3	*N* = 18
Kissing	2 (13%)	1	3 (17%)
Sexual Contact	11 (73%)	2	12 (67%)
Handshaking	0	0	0
I don’t know/other	3 (20%)	0	3 (17%)
*Heard of HPV Vaccine*	*N* = 40	*N* = 9	*N* = 49
Yes	10	3 (33%)	13 (26%)
No	30	6 (67%)	37 (74%)
Uncertain	0	0	0
*Believed the vaccine can be given to both males and females*	*N* = 10	*N* = 3	*N* = 13
Yes	7 (70%)	1	8 (61%)
No	2 (20%)	2	4 (31%)
Uncertain	1 (10%)	0	1 (8%)
*Believed the vaccine to be safe*	*N* = 10	*N* = 3	*N* = 13
Yes	8 (80%)	2	10 (77%)
No	1 (10%)		1 (8%)
uncertain	1 (10%)	1	2 (15%)
*Believed the vaccine was only for those who are sexually active*	*N* = 10	*N* = 3	*N* = 13
Yes	4 (40%)	1	5 (38%)
No	6 (60%)	1	7 (54%)
Uncertain		1	1 (8%)
*Believed the vaccine is effective in preventing certain types of cancers*	*N* = 10	*N* = 3	*N* = 13
Yes	5 (50%)	1	6 (46%)
No	3 (30%)	1	4 (31%)
Uncertain	2 (20%)	1	3 (23%)
*Believed the vaccine is most effective if given before one become sexually active*	*N* = 10	*N* = 3	*N* = 13
Yes	8 (80%)	2	10 (77%)
No	0		0 (0%)
Uncertain	2 (20%)	1	3 (23%)
*Believed the vaccine can cure the disease*	*N* = 10	*N* = 3	*N* = 13
Yes	4 (40%)	1	5 (38%)
No	4(40%)	2	6 (46%)
Uncertain	2 (20%)	0	2 (15%)
*Believed the vaccine protects against all sexually transmitted infections*	*N* = 10	*N* = 3	*N* = 13
Yes	3 (30%)		4 (31%)
No	6 (50%)	3	8 (61%)
Uncertain	1 (10%)		1 (8%)
*Worried that the vaccine can cause bad side effects*	*N* = 10	*N* = 3	*N* = 13
Yes	3 (30%)	1	4 (31%)
No	6 (60%)	2	8 (61%)
uncertain	1 (10%)		1 (8%)
*Believed that giving a child the vaccine will encourage them to have sex*	*N* = 10		*N* = 10
Yes	1 (10%)		1 (10%)
No	8 (80%)		8 (80%)
uncertain	1 (10%)		1(10%)
*Will you have your child vaccinated?*	*N* = 41		
Will be vaccinated	15 (36%)		
No, will not receive the vaccine	7 (17%)		
Undecided	13 (32%)		
Prefer not to say	6 (15%)		
*Will you or have you been vaccinated*		*N* = 6	
*vaccinated*		1 (17%)	
*not vaccinated*		3 (50%)	
*Undecided*		2 (33%)	
*Vaccinate child even if had to pay*	*N* = 36		
Yes	12 (33%)		
No	7 (19%)		
Uncertain	17 (47%)		
*Will you vaccinate your child if it was free/publicly funded?*	*N* = 36		
Yes	11 (31%)		
No	13 (36%)		
Uncertain	12 (33%)		
*Opinions of friends and family influence my decision*	*N* = 37	*N* = 2	*N* = 39
Yes	10 (27%)		10 (26%)
No	19 (51%)	2	21 (54%)
Uncertain	8 (22%)		8 (20%)
*Willing to get the vaccine if recommended by the doctor*	*N* = 41		
Yes	19 (46%)		
No	0 (0%)		
Uncertain	8 (19%)		
No response	14 (34%)		

Participants were able to choose one or more response in questions about HPV knowledge source and how HPV transmitted

Participants who answered yes to the question about whether they had heard of HPV were asked additional questions pertaining to their level of knowledge of HPV and the HPV vaccine. Fifteen out of 50 respondents had heard of HPV (28%) and 2 preferred not to say (4%). Of these, only 2 respondents’ source of knowledge was from either a Doctor or Nurse (14%). Other sources of information included family members (*n* = 2, 14%), friends (*n* = 1, 7%), school (*n* = 1, 7%), finding their own information (4, 43%) and ‘other’ (*n* = 2, 14%).

We asked the 15 participants who had heard of HPV questions about their HPV knowledge and 18 responses were provided. Twelve respondents (80% of respondents) knew that HPV is sexually transmitted. Of the 13 participants who had heard of the HPV vaccine, 5 (36%) believed that the HPV vaccine was only for those who are sexually active and only 8 (61%) believed that the vaccine was available for both men and women. Of the participants who had heard of the HPV vaccine, 6 participants (46%) knew that it is effective in preventing certain types of cancer. Nearly 40% (*n* = 5) incorrectly believed that the vaccine can cure HPV. Only 1 respondent answered all 6 questions correctly.

### HPV vaccine beliefs

Ten of the 13 participants (77%) who had heard of the HPV vaccine believed that the vaccine is safe, and 8 (61%) stated that they were not worried about its side-effects; however, when these individuals who had heard of the vaccine were asked about intent to vaccinate, less than two-thirds of these respondents said they would accept (or have already accepted) the HPV vaccine (*n* = 8, 62%).

Thirty-six out of the forty-one caregivers who completed the survey answered the question about discussing sexual health with their children. Only 16 of the 36 respondents (44%) reported feeling comfortable having these discussions. Among the caregiver population, 10 participants had heard of HPV (28%). 80% (*n* = 8) of caregivers who had heard of the vaccine believed that having their child vaccinated against HPV would not encourage promiscuity. Furthermore, all 8 of these participants who did not believe the vaccine promoted sexual behaviour also felt comfortable talking to their children about sexual health. However, during the qualitative interviews some parents reported feeling uncomfortable discussing sexual health with their children, especially with their daughters (Table 2). One participant was quoted as saying: *“I believe this is a bit difficult to speak about …*. *Africans don’t talk a lot about sex with their children. If it’s with girls, no it is difficult, with boys I can see us talking a little bit, but not in depth. With girls it is very difficult.”*

Young adults were asked whether they were comfortable talking about health-related issues including sexual health with their parents. Only 4 provided an answer to this question, of which 1 answered yes. All participants who chose not to answer this question were 21 years of age or older.

### Access and acceptance of vaccination

Willingness to vaccinate was not associated with region of origin, language, sex, level of education or newcomer status (e.g., immigrant, refugee). In terms of cost, one-third of respondents (*n* = 12) indicated that they would accept the vaccine even if they had to pay for it. The remaining two-thirds (*n* = 24) were either unsure whether they would accept the vaccine or stated that they would only accept the vaccine if it was free. One interviewee stated “*if it’s affordable, I really don’t mind because we are talking about life here”.*

Despite not previously hearing of HPV or the vaccine, many newcomers expressed willingness to have their children vaccinated or to be vaccinated themselves. Of the 41 participants who completed the caregiver survey, 15 (36%) indicated that they would have their children vaccinated against HPV, 19 (46%) preferred not to say or were undecided. There was an association between willingness to have their child vaccinated and level of education, although this did not reach significance (*X*^*2*^(4, *N* = 41)=8.9419, *p =* 0.063). Willingness of caregivers to have children vaccinated was not associated with caregiver age (*X*^*2*^(4, *N* = 41)=1.4418, *p =* 0.837) or gender (*X*^*2*^(2, *N* = 41)=1.566, 0.457).

Of the 26 individuals who decided not to vaccinate their child against HPV or had not yet decided, 18 (69%) said that they would accept the vaccine if it was recommended by a clinician. No participants indicated that they would definitely refuse the vaccine if a doctor recommended it.

*Interviewer: “If your doctor was to say, “I think that your child should be vaccinated.” Would that influence your decision”?*

*“It’s okay. I am sure the doctor will never mislead”.*

Only 3 survey respondents provided a response when asked why they would not have their child vaccinated. The following responses were provided: ‘*I would want to discuss it with her father’; ‘they will be educated about sexual health’; ‘my decision would depend on my doctor’s opinion. If he said to give it to my child, I would do it.’*

Of the 9 participants who completed the young adult survey, 6 answered the question about receiving the vaccine. Of these, 1 participant (17%) had already received the vaccine, 2 (33%) had not been vaccinated and 3 (50%) had not yet decided whether they would receive the vaccine.

Participants who completed the interviews were asked to give suggestions on how to inform newcomers to Canada about the HPV vaccine. Two participants suggested culturally- and language-appropriate materials should be made available in doctors’ offices or in the community and one participant suggested that newcomers should be educated during the immigration process.

## Discussion

This study sought to understand barriers to newcomers’ uptake of the HPV vaccine by surveying 50 individuals who self-identified as newcomers to Canada about their knowledge, attitudes, and beliefs regarding HPV and the HPV vaccine. The survey highlighted considerable gaps in newcomers’ awareness of both HPV and the vaccine, with most participants reporting never having heard of either. Among those who had heard of HPV and/or the vaccine, misconceptions were common.

The lack of knowledge about HPV demonstrated by the participants in our study is not unique, and has been documented among newcomers in a variety of contexts due to a combination of systemic and cultural factors [10, 19–21]. These issues of low HPV-knowledge also appeared in our work exploring healthcare providers’ perceptions about newcomer uptake of the HPV vaccine [22] and a systematic review of studies exploring barriers to vaccination faced by newcomers generally [14]. Newcomers often report a complete lack of awareness of the HPV vaccine [23, 24], as was the case for nearly three-quarters (74%) of participants in our current study.

**Fig. 1:**
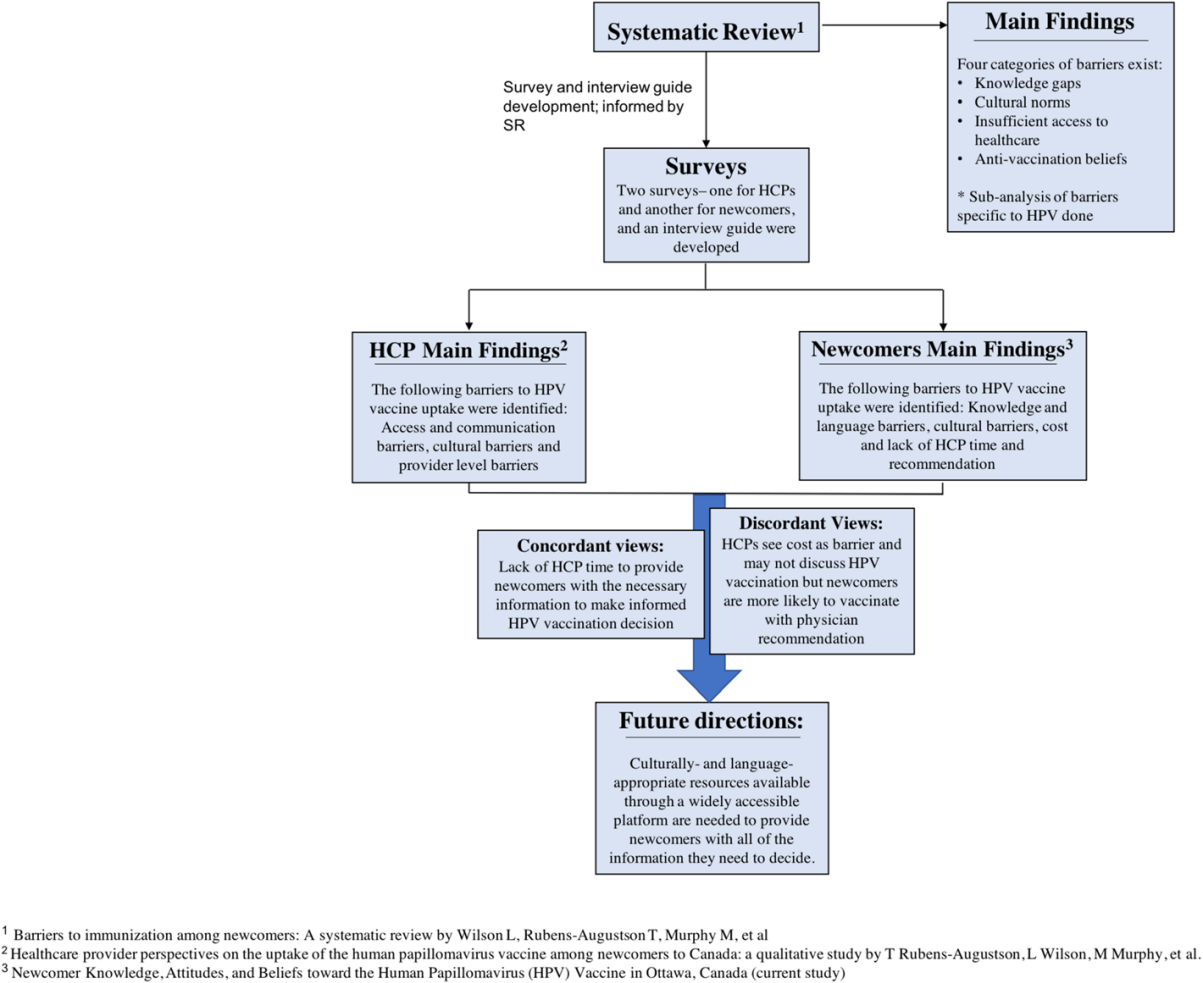
Consolidation of findings from barriers to HPV vaccination studies conducted by our research group

Cultural factors can also play a substantial role in newcomers’ HPV vaccine decision-making. Specifically, cultural taboos around discussing sexuality and concerns that the vaccine will promote promiscuity have been documented as important barriers for many newcomers [10]. For participants in our study who were already familiar with the HPV vaccine, this did not appear to be an issue, as those who felt comfortable discussing sexuality with their children did not believe the vaccine contributed to sexual activity. However, in the qualitative interviews, participants did report experiencing difficulty discussing sexuality with their children, particularly their daughters. See Fig. 1 for a full comparison and consolidation of the findings from our previous studies.

Vaccine hesitancy that is associated with deeply-held beliefs such as religion or culture can be very resistant to change. However, it is important to note that newcomers may be more receptive to vaccination overall than members of the general population [19] and that many participants in this study reported being undecided with respect to accepting the HPV vaccine for themselves or their children rather than opposed to it. Previous research has shown that a healthcare provider’s recommendation is a strong predictor of HPV vaccination and is crucial to increasing vaccine uptake [24, 25]. This finding is corroborated by the participants in our study, nearly half of whom indicated that their indecision could be overcome by a healthcare provider’s recommendation. Time spent by healthcare providers to fully engage with newcomers about HPV vaccination to provide information and/or clarify common misunderstandings or misconceptions about the vaccine is needed [21, 26] but not always feasible [22]. Here we also reported that only 2 participants had heard about HPV from a doctor or nurse. There is a need for culturally- and language-appropriate solutions that allow healthcare provider engagement and promote health literacy for newcomers without further taxing providers [27].

We reviewed the existing literature on barriers to HPV vaccine among newcomers and have summarized this in Table 4. These 9 studies identified a number of barriers contributing to low HPV vaccine uptake in immigrant populations that align with the findings of our study. Specifically, Yi et al. found that women who were less proficient in English were less likely to receive the HPV vaccine due to difficulty in understanding the educational material [28]. Participants whose cultural norms were taken into consideration were more likely to receive a vaccine [24, 29, 30]. Consistent with our current study, four studies identified a gap in healthcare provider recommendation and participants indicated that if their healthcare provider recommended it, they would consider getting the vaccine [20, 23, 30].

**Table 4:** Summary of barriers to HPV vaccination in newcomers

Reference	Study setting	Sample Size	Country of Origin/Ethnicity	Objectives	Methods	Results	Conclusion
Grandahl et al., 2015 [29]	Sweden	*n* = 50, women aged 18–54	Middle East, Africa, Asia, East Europe	To explore immigrant women’s experiences and views on the prevention of cervical cancer, screening, HPV vaccination and condom use.	8 focus group interviews, 5–8 /group	Emergent themes: 1) deprioritization of women’s health in home countries 2) positive attitudes of availability of women’s health in Sweden 3) positive and negative attitudes towards HPV vaccination 4) communication barriers limit health care access.	Women wanted to participate in cervical cancer prevention and would accept HPV vaccination for their daughters but faced barriers to information from HCP (language, cultural norms)
Aragones et al., 2017 [19]	USA	*n* = 36, parents of minors (i.e., 9–17 year old) who had not initiated the HPV vaccine series for their child	Latin America	To elucidate Latino immigrant parents’ barriers to obtaining the HPV vaccine for their children	5 focus groups, 5–10/group	Three major findings were: (1) low levels of awareness and knowledge of HPV and the HPV vaccine, (2) increased confidence that parent can access the vaccine for their eligible child and (3) lack of provider recommendation as the main barrier to vaccination.	Increased provider recommendation for the HPV vaccine while providing tailored HPV information to parents.
Stephens & Thomas, 2014 [30]	USA	*n* = 31, women, with a daughter between 11 and 18 years old	Haiti	To identify cultural beliefs influencing immigrant Haitian mothers’ willingness to vaccinate their daughters against HPV	Survey assessing HPV and HPV vaccine knowledge, followed by a semistructured interview.	Mothers had low levels of HPV and HPV vaccine knowledge, and asked for more information. Concerns centered on cultural values regarding adolescent sexuality and HIV/AIDS stigmas specific to Haitian communities.	Vaccination uptake could increase if recommended by a physician. Uptake efforts should emphasize physician involvement and incorporate culturally relevant health concerns.
Kobetz et al., 2011 [21]	USA	*n* = 41 women aged 21–75	Haiti	To examine Haitian women’s perceptions of, and barriers to, HPV vaccination	5 focus groups, 8/group	Amongst those participants who had heard of HPV, many held misconceptions about virus transmission and did not understand the role of HPV in the development of cervical cancer. All participants showed support for vaccines as beneficial for health.	Addressing gaps such as lack of educational information available in Haiti language about HPV and cervical cancer.
McComb et al., 2018 [10]	Canada	*n* = 11, women aged 18–26	Africa, Asia, South America	Exploring underlying reasons for lower uptake of (HPV) vaccine among new immigrants and refugees	Semi-structured interviews	Participants had limited knowledge about HPV and the HPV vaccine. Most women perceived that their risk of HPV was low, however showed willingness to receive the vaccine if it were recommended by their physician.	Efforts are required to increase knowledge about HPV among immigrant and refugee women and support for physicians to discuss and offer vaccination to this population.
Yi et al., 2013 [28]	USA	*n* = 113, women aged 18 or older	Vietnam	To determine receipt of HPV vaccine and assess if limited English proficiency and knowledge related to HPV vaccine were associated with HPV vaccine uptake	semi structured interviews	Women who were less proficient in English were less likely to receive the HPV vaccination. Participants lacked knowledge of HPV.	There is a need for public health evaluation and education programs on HPV and cervical cancer designed for Vietnamese-American women
Lee & Lee, 2017 [24]	USA	*n* = 16, women, aged 21 and older	Korea	This study aimed to identify major barriers to Papanicolaou (Pap) test uptake and HPV vaccine acceptability.	3 focus groups with 16 women	Three major themes emerged as barriers: 1) limited knowledge about cervical cancer and preventive behaviors, 2) culture-specific barriers, and 3) low accessibility to health care services.	Culturally tailored cervical cancer education is needed to promote Pap test uptake and HPV vaccination in this population.
Luque et al., 2010 [23]	USA	*n* = 80, women, aged 18 to 55 *n* = 17, HCW’s, average age 42.	Mexico, Hondurus, Peurto Rico, USA	To explore knowledge, attitudes, and beliefs regarding HPV, the HPV vaccine, and cervical cancerscreening	Surveys	Mexicans and Hondurans had different perceptions of risk factors and lower levels of HPV knowledge than Puerto Ricans or Anglo-Americans.	Target areas for health education based on resulting cultural models of illness need to be identified
Scarinci et al., 2007 [20]	USA	*n* = 55, women, aged 17 and 39 years old	Latinas and African Americans	To examine the acceptability of preventive HPV vaccination among Latina immigrants and African American women	8 focus groups were conducted	The motivating factors for vaccine use included (1) receiving education/information about the vaccine through healthcare providers, (2) affordable prices, (3) good results in trials, and (4) knowing others who had already gotten vaccinated.	These findings suggest that unique educational strategies need to be developed, based on the needs and perceptions of the targeted audience, in order to achieve wide-spread acceptability of this vaccine.

We identified 3 main strengths of this study. First, this study examined an issue of high public health importance and highlighted a need for health care engagement to promote HPV vaccine uptake in newcomer populations, which is supported by the literature. Second this study was conducted in a city with a high newcomer population, thus the outcomes we have identified are more likely to reflect the experiences of the broader newcomer population in Canada. Finally, the use of both quantitative and qualitative approaches allowed us to build upon survey findings.

A primary limitation of this study is its small sample size. Given the exploratory nature of this study, we relied on a convenience sampling approach, limiting generalizability. As our participants were recruited from CHCs in Ottawa, it is possible that our findings may not be applicable to other centers or other newcomer populations we did not reach. Additionally, because our participants already use the healthcare services offered by CHCs, it is plausible that these newcomers were more engaged in the Canadian healthcare system and may thus face different barriers than people who are less familiar with the health services offered for newcomers. Finally, because the study design was reliant on self-reported behaviours or intended behaviours, this study may suffer from issues of social desirability bias. As the study was conducted in CHCs that offer vaccination and given that participants knew that the nature of the study was to explore barriers to vaccination, they may have felt compelled to report higher levels of vaccine acceptance than they would have in other circumstances.

## Conclusions

Our study indicates that there are considerable gaps in newcomers’ awareness and understanding of HPV and HPV vaccination. Healthcare providers should seek to find ways to equip newcomers with culturally and language-appropriate resources to make informed vaccine decisions. A standardized approach could overcome language and communication issues and would allow for common information to be conveyed to patients. Future research should seek to engage a wider variety of newcomers in order to determine which barriers occur across newcomer populations and context.

## Supplementary Information

**Additional file 1: Table S1.** Qualitative Interview Guide.

## Data Availability

The datasets used and/or analyzed during the current study are available from the corresponding author on reasonable request.

## Abbreviations

HPV: Human papillomavirus
KAB: Knowledge, attitudes and beliefs
CHCs: Community health centres
