# Supplementary material for: Newcomer knowledge, attitudes, and beliefs about human papillomavirus (HPV) vaccination
Source: BMC Fam Pract. 2021 Jan 9;22:17. doi: 10.1186/s12875-020-01360-1 (PMC7797127; doi:10.1186/s12875-020-01360-1)
Supplement: Supplementary file 1 — Additional file 1: Table S1. Qualitative Interview Guide. [file 12875_2020_1360_MOESM1_ESM.docx]

Supplementary Table 1: Qualitative Interview Guide.

|  | **Question** | **Prompt** |
| --- | --- | --- |
| **Introduction** | *Let’s start by talking a little bit about you. Can you tell me about yourself?*    *Have you gotten any vaccines for yourself (or for your child) since arriving in Canada?* | · *How long have you lived in Canada?*  · *What country did you come from?*  · *Do you have any children?* |
| **HPV Vaccination** | *Have you heard of HPV before?*  · *(if YES) What do you know about it?*    *Have you heard of the HPV vaccine? Other names for it are Gardasil and Cervarix.*  · *(if YES) What do you know about it?* |  |
| **Uptake** | *Have you or your children ever received the HPV vaccine?*   - *(if YES) What made you decide to get (your children) vaccinated for HPV?* - *(if NO) Why did you decide not to accept the HPV vaccine (for your children)?*     *Did your doctor recommend the HPV vaccine for you (or your kids)?* | ·  *How did you feel about the doctor recommending this vaccine?*  · *Did your doctor explain the vaccine to you?*  · *Was there anything about the vaccine you would have liked to know that wasn’t explained?*  · *What did your doctor do well?*  · *What could your doctor have done better?* |
| **Promoting vaccination** | *Do you have any suggestions for how to encourage newcomers to get vaccinated against HPV?* |  |
| **Other** | *Is there anything else you’d like to talk about regarding HPV vaccination among newcomers?* |  |
